# Mice repeatedly exposed to Group-A *β*-Haemolytic Streptococcus show perseverative behaviors, impaired sensorimotor gating, and immune activation in rostral diencephalon

**DOI:** 10.1038/srep13257

**Published:** 2015-08-25

**Authors:** Simone Macrì, Chiara Ceci, Martina Proietti Onori, Roberto William Invernizzi, Erika Bartolini, Luisa Altabella, Rossella Canese, Monica Imperi, Graziella Orefici, Roberta Creti, Immaculada Margarit, Roberta Magliozzi, Giovanni Laviola

**Affiliations:** 1 Sect. Behavioural Neuroscience, Dept. Cell Biology & Neuroscience, Istituto Superiore di Sanità, Viale Regina Elena, 299, I-00161 Roma, Italy; 2 IRCCS-Istituto di Ricerche Farmacologiche “Mario Negri”, Via G. La Masa 19, 20156 Milano, Italy; 3 Research Centre, Novartis Vaccines and Diagnostics, Via Fiorentina 1, 53100 Siena, Italy; 4 Sect. Molecular and Cellular Imaging, Dept. Cell Biology & Neuroscience, Istituto Superiore di Sanità, Viale Regina Elena, 299, I-00161 Roma, Italy; 5 Sect. Respiratory and Systemic Bacterial Diseases, Dept. of Infectious, Parasitic, and Immune-mediated Diseases, Istituto Superiore di Sanità, Viale Regina Elena, 299, I-00161 Roma, Italy; 6 Sect. Demyelinating and Inflammatory Diseases of the CNS, Dept. Cell Biology & Neuroscience, Istituto Superiore di Sanità, Viale Regina Elena, 299, I-00161 Roma, Italy

## Abstract

Repeated exposure to Group-A *β*-Haemolytic Streptococcus (GAS) may constitute a vulnerability factor in the onset and course of pediatric motor disturbances. GAS infections/colonization can stimulate the production of antibodies, which may cross the blood brain barrier, target selected brain areas (e.g. basal ganglia), and exacerbate motor alterations. Here, we exposed developing SJL male mice to four injections with a GAS homogenate and evaluated the following domains: motor coordination; general locomotion; repetitive behaviors; perseverative responses; and sensorimotor gating (pre-pulse inhibition, PPI). To demonstrate that behavioral changes were associated with immune-mediated brain alterations, we analyzed, in selected brain areas, the presence of infiltrates and microglial activation (immunohistochemistry), monoamines (HPLC), and brain metabolites (*in vivo* Magnetic Resonance Spectroscopy). GAS-exposed mice showed increased repetitive and perseverative behaviors, impaired PPI, and reduced concentrations of serotonin in prefrontal cortex, a brain area linked to the behavioral domains investigated, wherein they also showed remarkable elevations in lactate. Active inflammatory processes were substantiated by the observation of infiltrates and microglial activation in the white matter of the anterior diencephalon. These data support the hypothesis that repeated GAS exposure may elicit inflammatory responses in brain areas involved in motor control and perseverative behavior, and result in phenotypic abnormalities.

A growing body of experimental and clinical evidence indicate that repeated infections may constitute a vulnerability factor for the development or exacerbation of neuropsychiatric disorders entailing compromised motor function^1^,^2^. The potential relationship between streptococcal infection and movement disorders has been observed in several cases of Tourette’s Syndrome (TS) and Sydenham chorea (SC)^3^. Cardona and Orefici observed remarkably higher levels of anti-streptococcal antibodies in a large cohort of TS patients compared to healthy controls^4^. Similarly, Rizzo and colleagues reported increased anti-streptococcal antibody titers in TS patients^5^, and Bombaci and collaborators reported that sera of patients with tic disorders exhibit immunological profiles typical of a broad, specific, and strong immune response against Group-A β-Haemolytic Streptococcus GAS^6^. An analogous pathogenic mechanism has been identified for SC, a neurological disorder characterized by involuntary movements associated with obsessive-compulsive symptoms and emotional liability^7^,^8^.

Concerning the potential link between peripheral infections and neurological disorders, several authors proposed that immunological adaptive responses towards specific pathogens might result, in vulnerable individuals, in maladaptive phenomena^2^,^9^. Specifically, antibodies produced in response to Streptococcus may, under conditions of increased permeability of the blood brain barrier, result in autoimmune responses directed toward specific brain targets^9^. In accordance with the observation of motor clinical symptoms, the molecular mimicry – and the resulting inflammatory processes – has been proposed to occur at the level of the basal ganglia, a brain structure directly involved in motor control^2^. Swedo and collaborators coined the acronym PANDAS to define these Pediatric Autoimmune Neuropsychiatric Disorders Associated with Streptococcal infections^2^,^10^. Dale and Brilot cogently discussed the possibility that autoimmune processes may occur more often in patients characterized by movement disorders than in the control population^11^.

Whilst streptococcal infections play a considerable role in exacerbating movement disorders, several other factors contribute to their etiology. Notwithstanding the presence of remarkable knowledge regarding the pathophysiological mechanisms, the pharmacologic treatment of choice is still constituted by neuroleptics (e.g. haloperidol and pimozide) both in TS^12^,^13^,^14^ and SC^15^,^16^,^17^. Just as symptoms’ severity peaks during puberty, so also pharmacological therapy is given during this highly plastic stage of individual development. Thus, the possibility that current treatment strategies may beget enduring developmental side effects is considerably high^13^. Given the multifactorial etiology of these disorders and the paucity of therapeutic approaches, it is crucial to develop animal models of PANDAS upon which testing alternative hypotheses regarding the potential regulatory role exerted by genetic and environmental factors, and/or innovative therapeutic approaches^18^.

Repeated immunizations with GAS homogenate have been previously reported to result in the exhibition of motor alterations and in immunohistochemical abnormalities in mice^19^,^20^ and rats^3^,^21^. Specifically, Hoffman and colleagues showed that immunization of female mice with GAS homogenates resulted in increased rearing behavior and that these alterations were associated with IgG deposits in deep cerebellar nuclei^19^; Yaddanapudi and colleagues provided support to the autoimmune explanation of these data through a passive immunization study^20^. Thus, they showed that passive transfer of antibodies produced by GAS-treated mice resulted in behavioral and immunohistochemical abnormalities similar to those observed in response to active immunization^20^. Finally, Brimberg and collaborators reported that exposure to GAS in rats resulted in behavioral manifestations reminiscent of SC, which were alleviated by the administration of haloperidol^3^.

Leveraging these studies, here we tested the hypothesis that repeated exposure to GAS may constitute a vulnerability factor in the onset of neurological motor disturbances^22^. To this aim, we exposed SJL mice to repeated subcutaneous injections with a GAS homogenate^20^ during development (between late infancy and young adulthood), and then evaluated their short- and long-term effects on the aforementioned behavioral domains. Although PANDAS occur in the pediatric population, technical constraints (see below for a detailed discussion) resulted in the necessity to evaluate the phenotypic alterations in young adult individuals. The relevance of the young maturational stage has been considered by beginning the injection protocol shortly after weaning. This study was aimed at extending existing literature^3^,^19^,^20^ through the analysis of a wider spectrum of variables compared to those previously considered. Thus, here we investigated whether exposure of developing mice to GAS homogenates resulted in inflammatory processes in several brain areas, we extended the analysis of brain parameters to magnetic resonance spectroscopy and neurochemistry, and performed a behavioral test battery encompassing most of the parameters often associated with PANDAS. Specifically, to address whether the phenotypic alterations were specific to repetitive behaviors and perseverative responding or generalized to other domains, we performed a series of tests evaluating general locomotion, motor coordination, anxiety, sensorimotor gating (pre-pulse inhibition, PPI) and stereotypies. To evaluate the functional state of core brain areas involved in PANDAS, we performed magnetic resonance spectroscopy (MRS) in the striatum and prefrontal cortex. In line with clinical data indicating that movement disorders are often associated with major alterations in brain monoamines^23^ and that PANDAS symptoms may be mitigated by the administration of selective serotonin reuptake inhibitors^3^,^24^,^25^ and dopaminergic antagonists^26^,^27^, we also measured concentrations of serotonin, dopamine, and their metabolites in hippocampus, striatum, prefrontal cortex, cerebellum and hypothalamus. The presence of GAS specific antibodies, in sera from mice repeatedly injected with GAS homogenates formulated in Freund’s or with adjuvant alone as negative control, was investigated by Western Blot analysis. Ultimately, to confirm a pathophysiological link between GAS exposure and the presence of autoantibodies directed against brain targets, we performed histochemical and immunohistochemical assessment of the potential presence of inflammatory conditions linked to the experimental model examined in the current work, with particular attention to the identification of the specific types of immune-mediated responses.

## Materials and Methods

### Animals and rearing conditions

All the experiments were conducted between February and June, 2013. Male SJL/J mice (post-natal day, (PND) 22) were purchased from Charles River, Italy (Calco, LC). Upon arrival, all mice (N = 100) were weighed and housed individually in standard polycarbonate cages (33.0 × 13.0 × 14.0 cm) with sawdust bedding and *ad libitum* water and food (Mucedola, Settimo Milanese, Italy). They were maintained in an air-conditioned room (temperature 21 ± 1 °C and relative humidity 60 ± 10%) with a reversed 12-h light-dark cycle (lights on at 19.00 h). All experimental procedures were performed in accordance with European Communities guidelines (EC Council Directive 86/609), Italian legislation on animal experimentation (Decreto L.vo 116/92) and NIH guide for the care and use of laboratory animals. The study has been approved by the Service for Biotechnology and Animal Welfare of the Istituto Superiore di Sanità and authorized by the Italian Ministry of Health (Decree Nr. 217/2010-B).

### Immunization Protocol

#### GAS Homogenate Preparation

Group A Streptococcus (GAS) homogenates used for mouse immunization procedure were prepared as described in^19^ and stored at −70 °C. A sample of the homogenate (2.5 μL) was used to inoculate a blood agar plate to verify that it contained no viable bacteria.

### Active immunization of mice with GAS homogenates

The immunization protocol, which entailed four injections interspaced by three weeks, followed the procedure described by Hoffman *et al*.^19^ and Yaddanapudi *et al*.^20^. The first injection was performed on PND 28. Each mouse was assigned randomly to one of two groups: control (CTRL) group (N = 48) and GAS group (N = 48). During the primary injection, CTRL mice were treated subcutaneously (s.c.) with 125 μL of an emulsion (1:1) containing PBS and Complete Freund’s adjuvant (CFA; Sigma Aldrich, Milano, Italy). Mice in the GAS group (N = 48) received (s.c.) with 125 μL of the same emulsion (CFA:PBS) containing 2.5 μL of GAS homogenate (0.52 mg/ml of total protein as determined by Bradford Assay, Biorad). All mice were then injected three additional times at 3-week intervals with 125 μL of an emulsion of phosphate buffered saline (PBS) and Incomplete Freund’s adjuvant (IFA; Sigma Aldrich, Milano, Italy) alone for CTRL group, or 125 μL of an emulsion (PBS:IFA) containing 2.5 μL of GAS homogenate for GAS group. The immunization protocol was meant to replicate the procedures adopted in the original articles^19^,^20^ upon which our study rests. Thus, beside the preparation of the homogenate and the doses administered, the time of first injection and subsequent boosts are similar to those adopted in the original studies. Time of first injection in^20^ varied between four and six weeks and subsequent boosts were administered at three-weeks intervals. In^19^, mice were first immunized at six weeks of age and then boosted three times at four or six-week intervals. To prepare the PBS/adjuvant emulsions we used the vortex method described by Flies and Chen^28^.

Blood (~100 μl) was collected by tail incision 14 days after the primary injection and after the first and second boosts. After collection, whole blood was allowed to clot at room temperature for 4–6 hours, then centrifuged at 3.000 rpm for 15 minutes. The serum supernatant was transferred into Eppendorf tubes and stored at −80 °C for the evaluation of antibodies.

### Behavioral testing

The first test battery was administered to a group of mice (Batch I, N = 10 per group) one week after the primary immunization (week 5). An independent group of mice (Batch II, N = 10 per group) were screened for the same test battery one week after the second boost (week 11). For each test battery, mice performed five different behavioral tests in the following sequence: *elevated 0-maze* and *dowel test* on the first day; *rotarod test* and *marble burying* on the second day; and *open-field* on the last day. On the morning of the test, mice were moved to a behavioral testing room where they remained until the end of the daily test session. A group of 20 mice from the third batch (Batch III) were then screened for perseverative behaviors in a *T-maze* one week after the second boost (week 11). Finally, a separate group of individuals (Batch IV) were tested for sensory-motor gating through the evaluation of PPI. All data have been analyzed by trained personnel blind to treatments.

#### Elevated 0-Maze

To evaluate the exploration of an environment imposing on the animal an approach-avoidance conflict, mice were tested on the elevated 0-maze^29^, a 5.5 cm wide circular runway made of black plastic with an outer diameter of 46 cm, and placed 40 cm above the floor. Two opposing sectors were protected by 16 cm high walls made of Plexiglas (closed sectors), and the two remaining sectors were unprotected (open sectors). Mice underwent the test after 1 hour of acclimatization in the experimental room between 11:00 and 15:00. The test was performed under dim lights. Each session lasted 5 minutes and started with the animal released in one of the closed sectors. Beside spatio-termporal variables (time spent and number of entries in each sector), the following behavioral responses were detected in frequency and duration: *rearing*, *grooming*, *stretched attend posture*, and *head dipping*. Time spent in the open sectors was considered an inverse index of anxiety state while the total number of entries was used as a measure of general activity (see Ref. 29 for additional details).

#### Dowel test

This test requires animals to balance on an elevated hardwood round dowel^30^ and is generally used to investigate motor coordination on a static apparatus. The dowel (0.9 mm in diameter and 35 cm long), covered with paper tape, was mounted horizontally 50 cm above the floor and fixed between two flat frames. A box filled with sawdust bedding was placed underneath the dowel to attenuate the fall. At the beginning of testing, each mouse was placed in the middle of the dowel so that the length of its body was parallel to the dowel. The time taken for the animal to fall from the dowel (latency to fall) was recorded. Cut-off time was 3 minutes. Each mouse was given three trials with 15 minutes intervals between them. Mice that walked across the dowel were assigned the cut-off time.

#### Accelerating rotarod test

The rotarod apparatus (Basile, Comerio, Italy) was used to evaluate the mouse balance and motor coordination measured by the latency to fall from the apparatus^31^. Differently from the Dowel, the rotarod test addresses individual coordination on a dynamic apparatus. Specifically, it consisted of a rotating cylinder (3 cm in diameter) on which the mouse had to continuously walk forward in order to avoid falling from the rod. The time spent on the rod was automatically recorded by a timer connected to a switch that stopped when the mouse fell. Two mice were tested simultaneously. Each mouse was given three trials with the rate of rotation of the rod increasing from 4 to 40 revolutions per minute (r.p.m.) over 4 minutes. Trials were interspaced by 10-min intervals. The trial ended when the mouse fell from the rod or reached the cut-off time (4 min). The falling latency averaged over three trials has been used as the dependent variable

#### Marble burying

The marble burying assay is often used as an indicator of anxiety-like behavior and/or obsessive-compulsive like behaviors^32^,^33^. Specifically, although initial burying episodes may connote a natural tendency exhibited by control animals, persistent burying bouts have been proposed to represent compulsive stereotypies dependent on a “frustrated investigation of the non-reactive stimulus-object”^32^. Testing was performed in standard polycarbonate cages (33.0 × 13.0 × 14.0 cm) filled with a 5-cm layer of sawdust bedding and covered with a wire mesh cage top without food and water. Twelve glass marbles (14 mm diameter) were evenly spaced in two rows on the bedding surface. Mice were placed individually in the cages and left undisturbed for a 30-min testing period. At the end of the test, each mouse was removed and the number of buried marbles was counted. A marble was considered buried when more than two-thirds of its surface was covered with bedding.

#### Open-field test

To evaluate exploratory activity and general locomotion, mice were tested in the open field. The apparatus consisted of a square arena (60 × 60 cm) with 60 cm high walls made of grey plastic. The floor of the apparatus was subdivided in 6 × 6 matrix (each square was 10 × 10 cm). A central square (20 × 20 cm) was drawn in the middle of the open field to delimitate the central area of the arena. The maze was located in the experimental room and lit by a 60-watt lamp for background lighting.

The test started by placing the mouse in one corner of the arena and lasted 45 min. The open field was cleaned between each session using 10% ethanol/water solution. The activity of mice during the test was video recorded with a video camera suspended from the ceiling. The behaviors expressed during the first and last five minutes of recording were subsequently scored by a trained observer using a computer with dedicated software (The Observer 2.0, Noldus Information Technology, Wageningen, The Netherlands). The behavioral responses scored included: *line crossing*, *rearing*, *sniffing*, *grooming*, and *immobility*. Each animal was given a score for total locomotor activity that was calculated as the sum of line crossings. Time spent in the central area of the arena was also measured as an inverse index of anxiety.

#### T-Maze

A third subset of animals (Batch III, N = 9–10 per group) was screened for perseverative behavior in the T-Maze. This test is traditionally used to investigate perseverative responses in laboratory rodents through the evaluation of two consecutive choices in a paradigm in which subjects are given two alternatives. While control subjects generally exhibit spontaneous alternations, perseverative individuals display an elevated rate of consecutive identical responses. The apparatus was an enclosed maze with the form of a T consisting of three arms of equal size (50 × 16 cm). The test was performed in the room where the animals were housed and comprised 10 trials performed during five consecutive days. In each session the mouse was placed in the starting compartment facing the wall of the apparatus. The mouse was allowed to explore the apparatus for 2 minutes. As soon as the animal entered (with all four paws) one of the two alternative arms (left or right) the door of that compartment was closed and that arm was indicated as the first choice of the animal. After few seconds, the animal was gently removed from the maze and replaced in the starting compartment to perform a second choice trial. Mice generally alternate their choice of goal arm on the second trial if it is performed shortly after the first one, a behavior called ‘spontaneous alternation’^34^. The percentage of arm alternations was recorded for each mouse.

#### Pre-pulse inhibition (PPI)

PPI is traditionally adopted to evaluate sensorimotor gating capabilities in mammals through the analysis of the reduction in startle response produced by the presentation of a prepulse^35^. The experimental apparatus (med associates inc. St Albans, VT, United States of America, see^36^ for details) was constituted by a platform with a transducer amplifier (PHM-250–60) and an acoustic stimulator (ANL-925). The apparatus was positioned in a foam-lined isolation chamber (ENV-018S). Dimmed lighting and ventilation were guaranteed by a red light and a fan positioned inside the chamber. To ensure that the experimental subjects remained on the platform, the latter was enclosed in a perforated compartment. Experimental data were acquired and analyzed through dedicated software (SOF-815).

#### Procedure

For habituation, mice were individually placed in the startle chamber and left undisturbed for 5 minutes. On the following day, mice were positioned inside the startle chamber and exposed to a continuous white noise (62 dB) for 5 minutes; following this acclimation, mice were exposed to 10 pulses of 120 dB interspaced by an average inter-trial interval of 15 seconds. Finally, an 8-min session started, consisting of 28 trials. Each trial started with a 50-ms null period, followed by a 20-ms pre-pulse noise burst of 67, 70, 73 or 76 dB. The delay between the pre-pulse and the startle (40-ms 120 dB white noise) was 100-ms. The experiment entailed the following types of trial: pre-pulse plus startle (four trials per pre-pulse intensity), prepulse alone (four trials per pre-pulse intensity), startle alone (four trials) and no stimulation (four trials). To prevent habituation, the inter-trial interval varied between 10 and 20 seconds. Galvanic response (dependent variable) was measured every millisecond for 65 ms after the onset of the startle. Pre-pulse inhibition was measured as: PPI = [(A − B)/A]*100, wherein A is the baseline Galvanic reflex in response to the startle stimulus alone, and B is the reflex in response to the startle in pre-pulse plus startle trials.

### Experimental design

The general experimental design entailed mice to be exposed to repeated behavioral testing, Magnetic Resonance Spectroscopy, blood sampling, and neurochemical and immunohistochemical assays. To avoid pseudo-replications and distribute mice across the different dependent measurements, while maintaining an elevated statistical power, we formed four independent batches of mice: batch I, comprising 10 GAS-treated (four subcutaneous injections interspaced by three weeks) and 10 control mice treated with Adjuvant alone (control group, hereafter CTRL); batch II, comprising 15 GAS-treated and 15 CTRL mice; batch III, comprising 15 GAS-treated and 15 CTRL mice; and batch IV, comprising eight GAS-treated and eight CTRL mice. To avoid potential litter effects, littermates were attributed to different test procedures.

**Figure 1:**
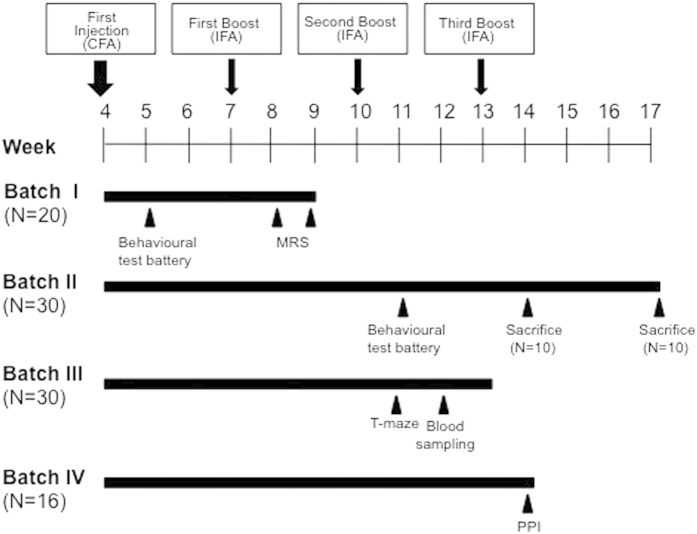
Experimental design. Timing of the injections expressed in weeks, and the test procedures performed on the four independent batches of mice. Animals received 4 doses of GAS homogenate, prepared as described in the Methods section, or Phosphate Buffer Saline (PBS), formulated with the indicated adjuvants (CFA = Complete Freund’s adjuvant; IFA = Incomplete Freund’s adjuvant).

Attribution of batches to experimental testing was designed as follows (see also Fig. 1):

*Batch I* comprised a total number of 20 animals (N = 10 per group) which encountered behavioral tests (*elevated 0-maze*, *dowel test, rotarod test, marble burying*, and *open-field* see below for details) and Magnetic Resonance Spectroscopy (MRS) analyses;

*Batch II* comprised 30 mice (N = 15 per group); 20 mice (N = 10 per group) encountered the same test battery of the batch I after the second boost; they were then sacrificed one week and four weeks after the third boost for monoamine analyses of specific brain areas (N = 5 per group) and brain IgG deposits evaluation and immunohistochemistry (N = 5 per group), respectively.

*Batch III* comprised a total number of 30 mice. We collected blood at different time points from 10 mice (N = 5 per group) for antibodies evaluation. A subset of animals, for a total number of 20 mice (N = 10 per group) was tested in a T-Maze to measure perseverative behavior.

*Batch IV* comprised a total number of 16 mice, which were used only for the analysis of pre-pulse inhibition (N = 8 per group), following the third boost.

### Magnetic resonance spectroscopy (MRS)

**Figure 2:**
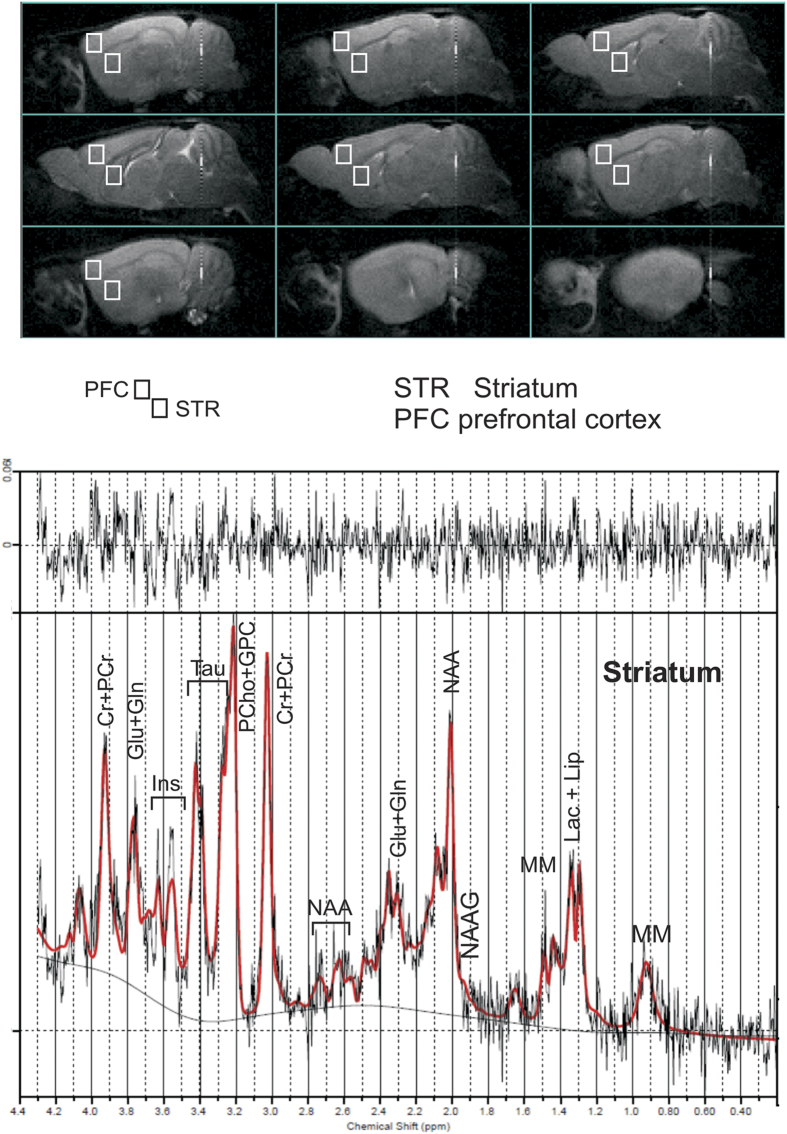
The upper part of the image represents the position of the two voxels on the Prefrontal cortex (PFC) and Striatum (STR) of sagittal anatomical images. The lower image is an example of the spectrum obtained from the analysis of the striatum (black trace) with overimposed the LCModel fit (red trace); it indicates the concentrations (ppm) of various metabolites. Metabolites which are included in the basis set: creatine (Cr), glutamate (Glu), glutamine (Gln), myo-inositol (Ins), lactate (Lac), N-acetylaspartate (NAA), N-acetylaspartylglutamate (NAAG), phosphocreatine (PCr), and taurine (Tau). Lipids (Lip) and macromolecules (MM) were also included in the basis set.

After the first boost, a group of mice (batch I, n = 7 per group; see Fig. 2) between 8 and 9 weeks of age underwent MRS analyses, with the aim of measuring the concentration of biochemical parameters in prefrontal cortex and striatum (see also^37^). All experiments were conducted on a 4.7 T Varian Inova animal system (Varian/Agilent Inc. Palo Alto, CA, USA), equipped with actively shielded gradient system (max 200 mT/m, 12 cm bore size) and a combination of volume coil for transmission and a surface coil as receiver (Rapid Biomedical, Rimpar, Germany). Further details on the apparatus are described in^38^. Single voxel localised ^1^H MR spectra (PRESS, TR/TE = 4000/23 ms, ns = 256 or 512) were collected from relevant brain areas: prefrontal cortex (PFC, 9.7 μl) and striatum (STR, 9.7 μl) (see Fig. 2). Quantitative MRS protocol, including water T2 measurements, was applied, see^39^ for details.

Spectra were analysed using LCModel^40^ that calculates the best fit to the experimental spectrum as a linear combination of model spectra (spectra of metabolite solutions). Further details on the methodology are described in Supplementary Information and in^38^.

### Brain sampling

For brain sampling, mice were rapidly decapitated; brains were removed and further processed for the subsequent analyses. Brain samples for immunohistochemical analyses were flash frozen and stored at −80 °C. Samples for evaluation of brain monoamines by HPLC analyses were immediately sectioned on ice to obtain prefrontal cortex, striatum, hippocampus, hypothalamus, and cerebellum sections. All samples were immediately flash frozen, and then stored at −80 °C until analysis.

### Immunohistochemistry

Brain samples have been collected after the third boost (week 14) from five GAS and five CTRL mice and immediately frozen in isopentane on dry ice. Coronal cryosections (10 μm thickness) were cut from whole brains and stored at −80 °C. Haematoxylin and eosin staining was performed on all the examined brain samples (1 cryosection every 15 cut cryosections) in order to assess the presence of potential inflammatory infiltrates. For immunohistochemistry assessment of level of microglia activation, air dried cryosections were passed in 70%, 95% and 100% ethanol, and after rehydratation with PBS and 20-min incubation with 0.3% H_2_O_2_ in PBS to eliminate endogenous peroxidase activity, sections were pre-incubated with 10% of normal donkey serum and incubated at 4 °C overnight with rabbit polyclonal antibody IBA1 (Wako**-**Chem, Japan) diluted in PBS containing 5% normal donkey serum, followed by incubation with biotinilated donkey anti-rabbit secondary antibody (Jackson Immunoresearch Laboratories, Suffolk, UK) visualized with the avidin-biotin horseradish peroxidase complex (ABC Vectastain Elite kit, Vector Laboratories, Burlingame, CA, USA) and 3,3 diaminobenzidine (Sigma Chemical, St. Louis, MO, USA). All sections were counterstained with haematoxylin, sealed with Canada balsam and viewed and photographed with an Axiophot Zeiss microscope (Germany) equipped with an Axiocam digital camera using the Axiovision 4 AC software. For immunofluoerescence, air-dried sections were pre-incubated with 10% of normal rabbit serum and incubated at 4 °C overnight with rat anti-mouse CD3 (Serotec, Oxford, UK) or anti-CD45R/B220 (Pharmingen, San Diego, CA, USA) antibodies, for T and B lymphocyets respectively, diluted in PBS containing 5% normal rabbit serum, followed by incubation with TRITC coniugated rabbit anti-rat secondary antibody (Jackson). Sections were sealed in ProLong Gold antifade reagent with 4’,6-diamidino-2-phenylindole (DAPI) (Invitrogen, Waltham, MA, USA), for the nuclei localization, and images were acquired and analysed with a Leica DM-4000B Microsystems microscope (Germany). Qualitative analysis of the presence and distribution of inflammatory cells was performed by a trained observer who did not perform the cutting (R.M.) blind to treatment across all the different brain areas.

### Monoamine measurements

Concentration of 5-HT, DA and their metabolites, i.e. 5-hydroxyindole acetic acid (5-HIAA), 3,4-dihydroxyphenylacetic acid (DOPAC) and homovanillic acid (HVA), were quantified by a modified method of HPLC combined with electrochemical detector (EC) as previously described^41^. Briefly, each brain region was weighed and a measured volume (10% W/V) of 0.1 N perchloric acid containing 0.05% Na_2_S_2_O_5_ and 0.1% Na_2_EDTA was added. The tissue was then disrupted by ultrasonication, centrifuged (10,000 × g; 5 minutes), and 100 μl of the supernatant were removed and filtered through 0.45 μm PVDF syringe filters (Perkin-Elmer, Italy). Aliquots of 20 μl were injected directly onto HPLC/EC system by a refrigerated (5 °C) autosampler (MIDAS, Spark-Holland, The Netherlands) for the separation of 5-HT, DA, 5-HIAA, DOPAC, and HVA through a Supelcosil LC-18DB, 3 μm (75 × 3.0 mm) analytical column (Supelchem, Italy), thermostated at 40 °C. The mobile phase consisted of sodium acetate anhydrous (5 g/L), citric acid (4.5 g/L), sodium octan sulphonate (100 mg/L), Na_2_EDTA dihydrate (112 mg/L); CH_3_OH 7%. Monoamines and metabolites were measured by a Coulochem II electrochemical detector (Dionex, Switzerland) equipped with a 5011 electrochemical cell (E1 and E2 potentials were 0 and +350 mV, respectively). A data system (Azur 4.6, Datalys, France) was used to calculate the concentration of analytes based on calibration curves prepared daily with appropriate concentrations of pure standards.

### Analysis of anti-Group A Streptococcal antibodies in serum samples

GAS homogenates obtained as described above were size-separated by SDS-PAGE (4–12% acrylamide) under reducing conditions and electroblotted onto nitrocellulose membranes. Immunostaining was performed by blocking the membrane overnight with 3% (w/v) skimmed milk in TPBS (0.1% Tween in PBS) and incubating for 2 h with sera from CTRL mice treated with adjuvant alone and from GAS-treated mice (all sera were diluted 1: 200). After 3 washes with TPBS, the membrane was incubated with HRP-conjugated secondary antibody (1:1000), washed again with TPBS and PBS, and developed with a chromogenic substrate.

### Statistical analysis

All statistical analyses were conducted using the software Statview 5.0 (Abacus Concepts, USA). Since the experimental design entailed two groups of independent observations (CTRL and GAS subjects), experimental data have been analyzed through t-test for independent samples (i.e. analysis of variance – ANOVA – for two groups). Situations in which repeated observations were collected on the same subjects were analyzed through repeated measures ANOVA for split-plot designs. Thus, group comparisons of the behavioral responses in elevated 0-Maze test and in T-maze were performed using t-test, with treatment (CTRL *vs* GAS) as the between-subjects factor. The statistical model for Dowel test, rotarod test and Marble Burying was as follows: 2 treatments (CTRL *vs* GAS) × 3 trials (first, second, third) with treatment as the between-subjects factor and trial as the within-subjects factor; to investigate the effects of repeated immunizations the general model was: 2 treatments (CTRL *vs* GAS) × 2 sessions (week 5 *vs* week 11). Additionally, in the Dowel test, to evaluate the performance before and after the treatment, we also performed a t-test on the difference in falling latency exhibited on week 5 and week 11. The statistical model for open field was: 2 treatments (CTRL *vs* GAS) × 2 time bins (first and last five minutes). PPI data were analyzed through repeated measures ANOVA, wherein treatment (CTRL *vs* GAS) constituted a between subject factor and prepulse intensities (67, 70, 73 and 76 dB) a within-subjects factor. In the MRS analysis, we applied a t-test with treatment (CTRL *vs* GAS) as between-subjects factor for the analysis of each metabolite in each specific brain area. Mann-Whitney U-tests were used in situations in which ANOVA assumptions were not met. Brain monoamines were analyzed through one-tailed T-tests with treatment (CTRL *vs* GAS) as between-subject factor. Finally, immunohistochemical observations – qualitatively measured as presence or absence of infiltrates and microglial activation – were analyzed through Chi-square tests. Fisher’s protected least-significance difference (PLSD) test was used for *post-hoc* comparisons. The relationship between behavioral data and brain neurochemistry (5-HT concentration in hippocampus and 5-HIAA/5-HT turnover in cerebellum) was investigated through simple regression analysis. Data are always expressed as mean ± SEM. Statistical significance was set at *p* < 0.05.

## Results

### Evaluation of anti-GAS antibody responses in sera from mice treated with GAS homogenates

**Figure 3:**
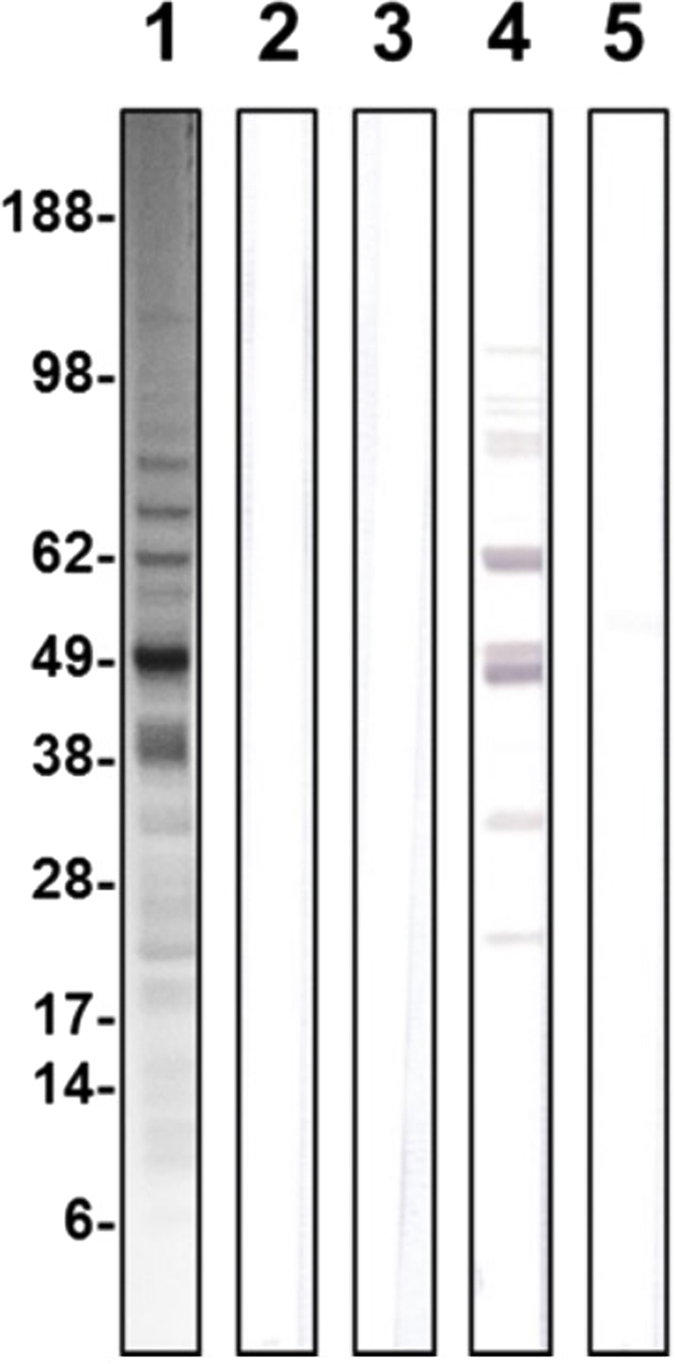
Western Blot analysis of GAS extracts probed with pooled sera from mice treated with GAS or with Adjuvant as Control. Lane 1, Coomassie staining of GAS homogenates; lane 2, Western Blot results after probing with sera of mice injected with one dose of GAS homogenates from GAS treated mice; lane 3, Western Blot results after probing with sera of control mice injected with one dose of Adjuvant alone; lane 4, Western Blot results after probing with sera of mice injected with four doses of GAS homogenates from GAS treated mice; lane 5, Western Blot results after probing with sera of Control mice injected with four doses of Adjuvant alone.

To investigate the presence of GAS-specific antibodies in sera from treated mice, 10 μl of GAS homogenates were loaded onto SDS-PAGE, transferred to nitrocellulose, and tested with pools of sera from animals injected with one or four doses of GAS homogenates or adjuvant alone. As expected, Coomassie stained lanes displayed a complex pattern of proteins, typical of GAS homogenates (Fig. 3, lane 1). Western Blot analysis of these extracts using sera from animals treated with one dose of GAS homogenate or adjuvant alone did not reveal any band (Fig. 3, lanes 2 and 3 respectively). Several bands were instead detected in the lane incubated with sera from animals receiving 4 doses of GAS homogenates, but not with four doses of adjuvant alone (Fig. 3, lanes 4 and 5 respectively). The pattern in lane 4 pattern reflected the Comassie stained pattern of lane 1, except that only a subset of the bands present in lane 1 were also present in lane 4. In particular, we observed 1 faint band above 98 Kda, 4–5 faint bands between 62 and 98 kDa, 1–2 main bands just below 62 kDa, 2 of about 49 kDa, 1 between 28 and 38 kDa, and 1 between 28 and 17 kDa. The results indicate that sera from GAS treated mice recognized some of the proteins present in the GAS homogenate.

### Body weight gain

To evaluate whether repeated GAS injections resulted in differences in growth curves, we evaluated individual body weight gain throughout the entire experiment. We observed that CTRL and GAS mice showed an indistinguishable body weight gain throughout the study (treatment: *F*(1, 39) = 0.04, *p* = 0.9, data not shown).

### Elevated 0-Maze (E0M) test (week 5, first injection and week 11, boost 2)

**Figure 4:**
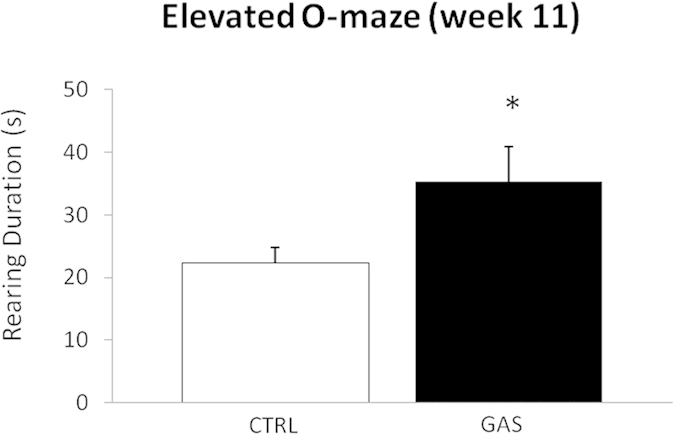
Rearing behavior in the elevated 0-maze (week 11, boost 2). The graph shows the duration of rearing behavior (s) in the elevated 0-maze during a 5-minutes long test session performed after the second boost (week 11). GAS mice significantly differ from CTRL mice. *p < 0.05. N = 10 per group.

We evaluated the behavioral profile of naïve subjects on the E0M test after both the first injection (week 5) and the second boost (week 11). Average values and standard errors of all observed data are reported in the supplementary material (see Table 1SM). As expected, experimental subjects showed a remarkable preference for the closed than the open sectors of the maze, whereby they spent approximately 85% of their time in the former and 15% in the latter. Following the first injection, CTRL and GAS mice did not show significant differences in general locomotion (frequency of entries, treatment: *F*(1,18) = 0.01, *p* = 0.93), time spent in the open arms (treatment: *F*(1,18) = 0.01, *p* = 0.91), and the ethological measures considered (duration of *rearing*, treatment: *F*(1,18) = 3.6, *p* = 0.07; frequency of *head dipping*, treatment: *F*(1,18) = 0.2, *p* = 0.7; frequency of SAP, treatment: *F*(1,18) = 0.4, *p* = 0.5; *grooming*, treatment: *F*(1,18) = 0.2, *p* = 0.6). Yet, following the second boost, while all mice spent almost the same amount of time in the open arms (11–12% of the single 5-minutes session, treatment: *F*(1,18) = 0.09, *p* = 0.8), GAS mice spent more time *rearing* compared to CTRL mice (treatment: *F*(1,18) = 4.54, p = 0.04, see Fig. 4). We did not observe significant between-group differences in the other behavioral parameters considered (frequency of *head dipping*, treatment: *F*(1,18) = 1.0, *p* = 0.3; frequency of SAP, treatment: *F*(1,18) = 1.5, *p* = 0.2).

### Dowel test (week 5, first injection and week 11, boost 2)

**Figure 5:**
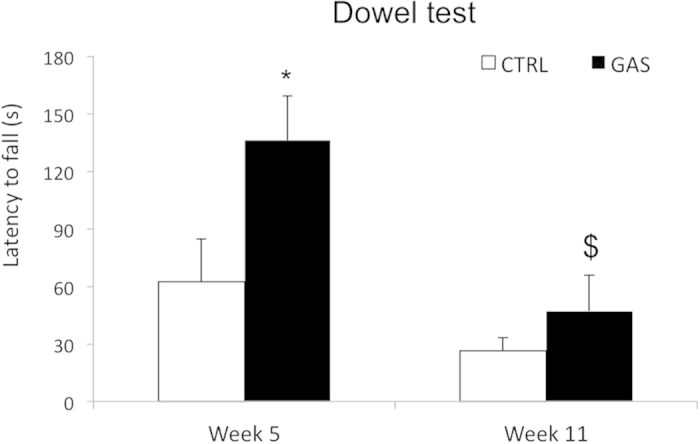
Motor coordination abilities in the dowel test. Falling latency (s) from a dowel covered with paper tape after the first injection (week 5) and the second boost (week 11). Histograms represent the average of three consecutive trials interspaced by 15-min intervals. *p < 0.05 in post-hoc tests between PBS and CTRL subjects; ^$^p < 0.05 significantly different from GAS mice in post-hoc tests performed between data observed on week 5 and week 11. N = 10 per group.

Following the first injection, compared to CTRL mice, GAS individuals spent more time on the dowel (treatment: *F*(1,18) = 5.3, *p* = 0.03). The overall performance of CTRL and GAS mice declined with time: thus, following the second boost, all subjects showed a shorter fall latency than that observed following the first injection (time: *F*(1,18) = 14.0, *p* = 0.001). However, the decline in performance was more pronounced in GAS than in CTRL mice (p < 0.05 in post-hoc tests, see Fig. 5).

### Rotarod test (week 5, first injection and week 11, boost 2)

All mice showed a general decline in motor coordination on the rotarod between the first injection and the second boost. Thus, all mice showed a shorter falling latency on week 11 than on week 5 (time: *F*(1,18) = 18.2, *p* = 0.0005). Additionally, CTRL and GAS mice showed an indistinguishable performance on the rotarod following both the first immunization (treatment: *F*(1,18) = 0.18, *p* = 0.68) and the second boost (treatment: *F*(1,18) = 0.4, *p* = 0.6, data not shown).

### Marble Burying (week 5, first injection and week 11, boost 2)

CTRL and GAS mice buried an indistinguishable number of marbles following both the first injection (treatment: *F*(1,18) = 1.8, *p* = 0.2; treatment × time: *F*(2,36) = 1.5, *p* = 0.2) and the second boost (treatment: *F*(1,18) = 0.05, *p* = 0.8; treatment × time: *F*(2,36) = 0.4, *p* = 0.7). However, the number of marbles covered on week 11 were remarkably higher than those covered on week 5 (time: *F*(1,18) = 8.6, *p* = 0.009). Specifically, during the first test battery CTRL mice covered 4 ± 0.7 marbles and GAS mice covered 2.9 ± 0.5 marbles. On week 11, CTRL mice covered 7.5 ± 1 marbles and GAS mice covered 8.3 ± 0.7.

### Open-field (week 5, first injection and week 11, boost 2)

Following the first injection, CTRL and GAS mice showed an indistinguishable performance on the open field test. Specifically, they spent a similar amount of time in the centre of the arena (treatment: *F*(1,18) = 0.019, *p* = 1.0), they showed indistinguishable levels of general locomotion (frequency of crossing, treatment: *F*(1,18) = 0.01, *p* = 0.9) and of *rearing* and *grooming* behavior (treatment: *F*(1,18) = 0.005, *p* = 0.9; and treatment: *F*(1,18) = 1.4, *p* = 0.3; respectively).

**Figure 6:**
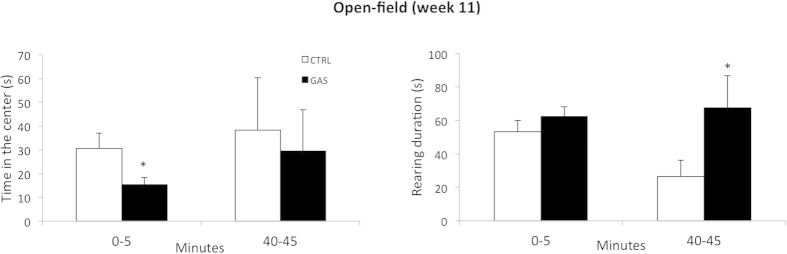
Open-field (week 11, boost 2). **(left)** Time spent in the center of the arena during the first and last five minutes of session recording; **(right)** duration of rearing behavior during the first and last five minutes of session recording; *p < 0.05 in post-hoc tests; N = 10 per group.

Following the second boost (week 11), compared to CTRL group, GAS mice showed a differential behavior on the open field. Thus, in the absence of major differences in absolute levels of locomotion (treatment: *F*(1,18) = 0.9, *p* = 0.4), during the first five minutes of testing, GAS mice spent remarkably less time in the center square of the arena compared to CTRL group (time: *F*(1, 18) = 290.1, *p* < 0.0001; treatment: *F*(1, 18) = 4.4, *p* = 0.05, see Fig. 6, left panel). During the last five minutes of the test session, GAS mice spent more time *rearing* compared to CTRL mice (treatment: *F*(1, 18) = 4.7, *p* = 0.04, see Fig. 6, right panel). Finally, irrespective of treatment (treatment: *F*(1,18) = 1.3, *p* = 0.3), all subjects spent more time *grooming* during the last five minutes of observation than during the first (time: *F*(1, 18) = 100.1, *p* < 0.0001).

### T-Maze

**Figure 7:**
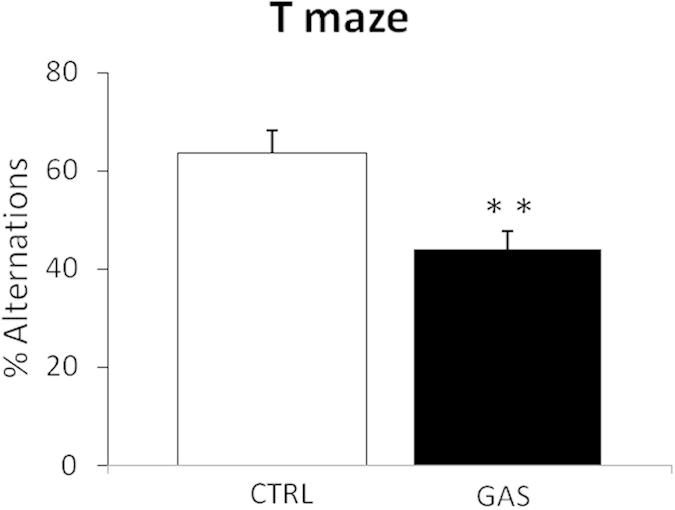
Spontaneous alternation in a T-maze (week 11, boost 2). Percentage of spontaneous alternations following the second boost (week 11). Values are expressed as mean ± SEM. **p < 0.01. N = 10, CTRL group; N = 9, GAS group.

The percentage of arm alternations was calculated as an inverse measure of perseverative behavior. GAS mice showed a significantly lower percentage of alternations compared to CTRL mice (treatment: *F*(1,17) = 11.0, *p* = 0.004, see Fig. 7).

### Pre-pulse inhibition

**Figure 8:**
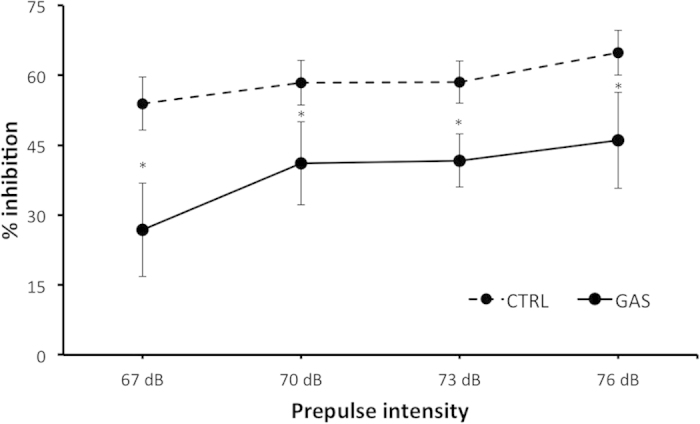
Sensorimotor gating measured through PPI (week 14, boost 3). Average inhibition of the startle reflex to a 120-dB stimulus following the presentation of a pre-pulse of different intensities (67, 70, 73, and 76 dB). Values are expressed as mean percentage PPI (%PPI) + SEM. *p < 0.05. N = 8 per group.

All subjects showed a reduced startle reflex in response to the pre-pulse plus pulse trials than to pulse alone. The percentage inhibition varied between groups (treatment: *F*(1,14) = 5.4, *p* = 0.036) with GAS individuals displaying reduced PPI compared to control individuals (see Fig. 8). Additionally, the different pre-pulse intensities induced a differential response (prepulse intensity: *F*(3,42) = 3.8, *p* = 0.016), with 67 dB eliciting the lowest response compared to the 70, 73, and 76 dB. However, this differential response was similar in CTRL and GAS individuals (treatment × prepulse intensity: *F*(3,42) = 0.57, *p* = 0.64). Ultimately, the two groups showed an analogous response to the pulse-alone trials (treatment: *F*(1,14) = 2.4, *p* = 0.143).

### Magnetic resonance spectroscopy

**Figure 9:**
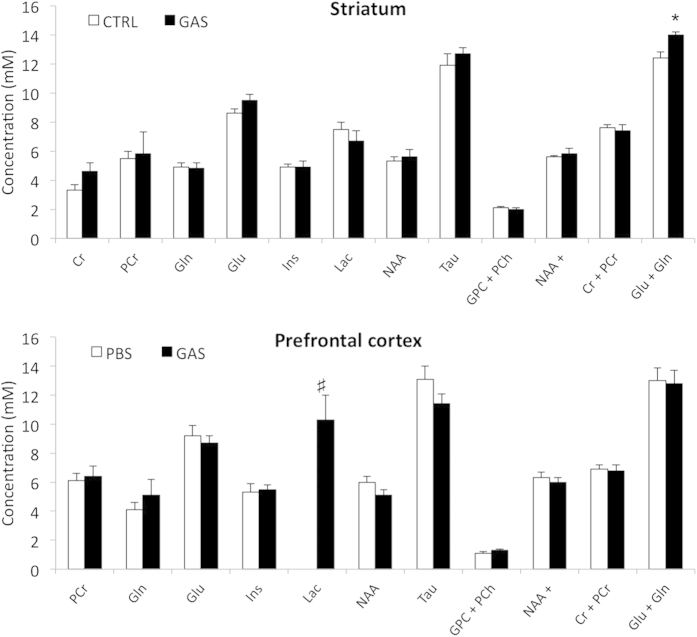
Mean levels of the metabolite concentrations (expressed in mM) obtained from *in vivo*^1^H spectra. **(Upper panel)** Striatum of CTRL and GAS groups. **(Lower panel)** Prefrontal cortex of CTRL and GAS groups. Data are expressed as mean ± SEM. *p < 0.05 in post-hoc tests performed between GAS and CTRL subjects. ^#^*p* < 0.01 in non-parametric test performed between CTRL and GAS mice.

^1^H MRS analyses at 4.7 T detected a few differences in the spectra acquired in the striatum and the PFC between CTRL and GAS mice (see Fig. 9). The quantitative results of all metabolite concentrations are reported in Fig. 9. In the striatum, we observed that GAS mice exhibited increased levels of the glutamate + glutamine pool (Glu + Gln) (*F*(1,10) = 13.4, *p* = 0.004). In the PFC, the analysis of the concentration of lactate revealed that GAS group had a remarkably high concentration of this metabolite compared to CTRL group. With respect to the latter, lactate was not quantifiable in any of the subjects tested. For this reason, we performed a non-parametric statistical analysis which revealed a significant difference between GAS and CTRL individuals (Chi^2^ = 3.48, N = 10, p < 0.01).

### Analysis of brain monoamines

The data regarding concentrations of monoamines and their metabolites in selected brain areas are reported in the supplementary material (see Table 2SM). We observed significant differences in serotonergic transmission in PFC and cerebellum. Thus, compared to CTRL, GAS mice exhibited reduced concentrations of 5-HT in the PFC (*F*(1,8) = 4.0, *p* = 0.04; 825.37 ± 94.13, and 615.68 ± 45.96 ng/g tissue weight for CTRL and GAS respectively) and increased serotonin turnover (5-HIAA/5-HT ratio) in the cerebellum (*F*(1,8) = 4.8, *p* = 0.03; 1.16 ± 0.09 and 1.35 ± 0.06 for CTRL and GAS respectively). We then addressed whether these findings correlated with the behavioral data observed in the same individuals (see Table 3SM for details). With respect to 5-HT concentrations in the hippocampus, we observed that while they did not relate to behavior in the elevated 0-maze, they significantly correlated with several behavioral indices exhibited in the open field: frequency of crossings (r^2^ = 0.50, p < 0.03); frequency of rearing (r^2^ = 0.52, p < 0.02); rearing duration (r^2^ = 0.60, p < 0.01); grooming duration (r^2^ = 0.65, p < 0.01). Additionally, although not significant, hippocampal serotonin apparently correlated with the latency to fall in the Dowel and Rotarod tests (r^2^ = 0.36, p = 0.06; r^2^ = 0.30, p = 0.09). Finally, 5-HIAA/5-HT turnover was correlated with the rearing behavior exhibited in the elevated 0-maze (r^2^ = 0.52, p < 0.02).

### Immunohistochemistry

**Figure 10:**
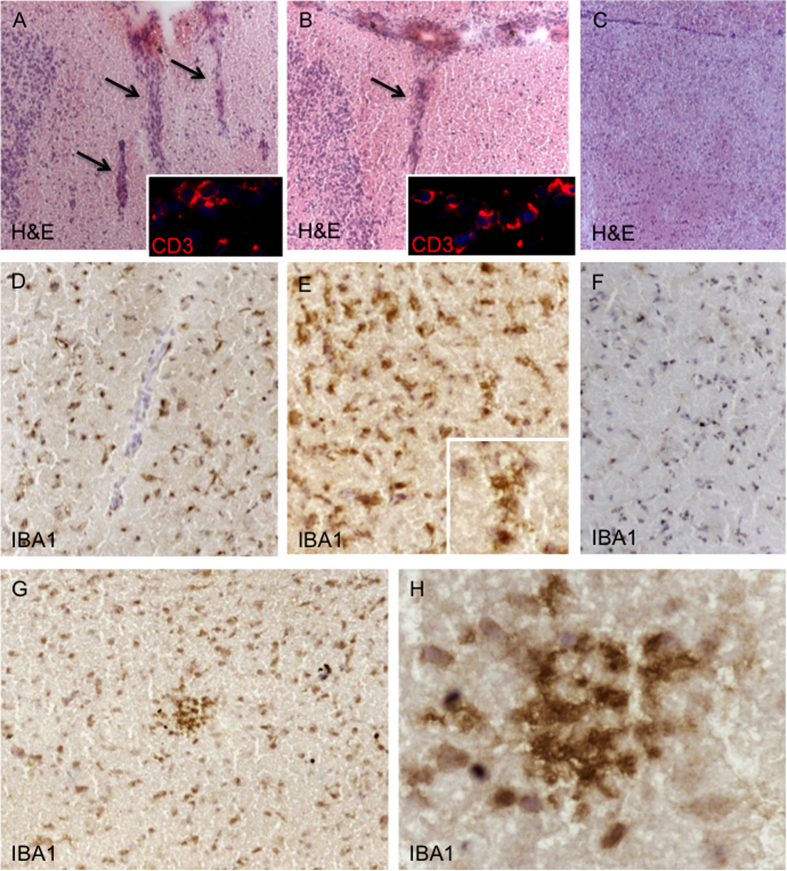
Neuropathology assessment of brains from GAS and CTRL mice. Panels (**A**,**B**) show inflammatory infiltrates (black arrows) in the white matter of the rostral diencephalon of two representative GAS mice, mainly containing CD3+ T lymphocytes (inset in (**A**,**B**)), not detected in the brains of control mice (**C**). Increased density of IBA1+ cells, morphologically resembling activated ramified microglia (inset in (**E**)), has been detected both around inflammatory infiltrates (**D**) and diffusely (**E**) in the mesencephalon of all the examined GAS mice, but not in the control mice (**F**). In some GAS mice, a group of IBA1+ activated microglia, forming a dense nodule, was found in the white matter in the rostral diencephalon ((**G**), higher magnification in (**H**)). Original magnification: 5X (**A**–**C**,**G**), 10X (**D**–**F**), 20X (inset in (**E**)), 40X (inset in (**A**,**B**)).

As reported in Fig. 10, histochemical and immunohistochemical investigations showed that, compared to CTRL mice, GAS mice exhibited indices of brain infiltrates (Chi^2^ = 3.26, N = 10, p < 0.05), associated with increased microglial activation (Chi^2^ = 3.06 N = 12, p < 0.05). In particular, scattered small inflammatory infiltrates (Fig. 10A,B), mainly containing CD3+ T lymphocytes (inset in Fig. 10A,B), were found in the white matter of the rostral and caudal diencephalon regions and more sporadically in the periventricular areas of GAS mice but not CTRL mice. Substantial increase in density of Iba1+ cells with morphology of ramified activated microglia has been detected both around the small inflammatory infiltrates (Fig. 10C) and, diffusely, across the white matter portion of both diencephalon and mesencephalon regions of all of the 5 examined GAS mice (Fig. 10D), but not in the CTRL (Fig. 10E). Moreover, nodules of IBA1+ activated microglia were found in scattered areas of the white matter of the rostral diencephalon in several of the examined GAS mice, without evidence of presence of any inflammatory infiltrate (Fig. 10F,G).

## Discussion

In the present study, we observed that repeated exposure of mice to a GAS homogenate, against which treated mice exhibited an antibody-mediated response, results in remarkable behavioral and neurological alterations. Thus, compared to control animals, GAS treated mice showed impaired sensorimotor gating, exhibited repetitive and perseverative behaviors at a higher rate, brain immunohistochemical and metabolic indicators of inflammatory processes, and alterations in monoamine concentrations. Importantly, the behavioral alterations were observed following repeated boosts and not in response to the first injection. This aspect, coupled with the immunohistochemical findings, strengthens the view that the observed phenotype may be associated with autoimmune responses to repeated exposure to GAS, that, in turn, target selected brain areas^9^,^19^. This hypothesis is substantiated by independent experimental evidence showing that serum obtained from GAS subjects reacts with rodent brain antigens and with human dopamine D1 and D2 receptor antigens^3^.

The behavioral alterations observed in GAS-treated mice are in accordance with the hypothesis that repeated exposure to streptococcus may relate to motor disturbances. Thus, the repetitive and perseverative behaviors shown at an abnormal rate by GAS treated mice are homologous to symptoms observed in PANDAS (e.g. obsessive-compulsive behaviors, tics and impaired sensorimotor gating^42^,^43^). The marked increment in the frequency of rearing behavior displayed by GAS treated mice may resemble human tics whereby it is invariant in its appearance, interrupts the regular behavioral routine, and is context-independent, as it is shown both in the elevated-0-maze and in the open field. Under normal conditions, rearing constitutes an explorative behavioral pattern regularly exhibited in a novel environment, which steadily declines with the progress of the experimental session^44^. However, when exhibited at an abnormal rate, such behavior may be the resultant of dysfunctional brain mechanisms. For example, excess rearing behavior has been observed in lesion studies involving the basal ganglia^45^ and in pharmacological studies interfering with dopaminergic and serotonergic transmission^46^,^47^. Additionally, present data replicate previous observations conducted in response to repeated GAS exposure^19^,^20^. In our study, the possibility that rearing constitutes an abnormal pattern rather than a functional response to a novel environment is supported by the temporal distribution in which it has been observed. Thus, in the open field test, control animals generally display elevated levels of rearing behavior during the early phases of the test and not towards the end^44^. Conversely, GAS treated mice spontaneously exhibited this behavior even 45 minutes after the beginning of the test session. In contrast with previous literature^20^, reporting a deficit in motor coordination in GAS mice, we failed to observe major alterations on the rotarod between GAS and PBS mice. Although we cannot fully explain such discrepancy, we offer that it may be due to the fact that while in the present study we evaluated male mice, Yaddanapudi and collaborators (2010) tested both male and female subjects. Thus, previous studies demonstrated that male and female mice may show a differential susceptibility to experimental treatments modulating motor coordination^48^. Furthermore, this differential sensitivity to experimental treatments has been observed in SJL mice^49^. Finally, our data are apparently conflicting with those reported by^3^ who observed variations in grooming behavior in GAS-treated individuals. In this case, the inconsistency is most likely due to procedural differences: specifically, while we investigated spontaneous grooming, Brinberg and collaborators (2012) assessed this behavior in response to the puffing of misted water. Additional procedural differences include the use of *Bordetella pertussis* (present in Brinberg *et al*., 2012 and absent herein) and the experimental species (rats vs. mice).

Beside abnormal repetitive behavior, GAS treated mice exhibited an elevated rate of perseverative responses in the spontaneous alternation test. The spontaneous alternation, generally exhibited by control mice in this test, has been proposed to reflect a natural tendency to explore the entire environment^34^. Such behavior entails the integrity of several brain structures including prefrontal cortex and dorsal striatum^50^ and is sensitive to the administration of dopaminergic^51^ and serotonergic^52^ drugs. The deficit observed in sensorimotor gating constitutes additional evidence in support of the hypothesis that repeated exposure to GAS may result in a dysfunctional regulation of forebrain circuitry^42^,^53^. Thus, PPI in laboratory rodents is remarkably reduced by the experimental lesion of striatal circuits^53^,^54^, and is modulated by the administration of dopaminergic drugs^55^,^56^,^57^,^58^,^59^. Additionally, antipsychotics have been shown to modulate PPI in schizophrenic patients^60^,^61^ and healthy controls^62^. Based on neuroanatomical, pharmacological, and clinical evidence, PPI has been associated with several disturbances including schizophrenia, obsessive-compulsive disorders, and TS (see Ref. 42 for a review).

Finally, rather than reflecting a generalized response, the phenotype exhibited by GAS treated mice appears specific to repetitive and perseverative behavior. Thus, GAS and CTRL mice showed an indistinguishable phenotype in anxiety-related tests, motor coordination and general locomotion. These considerations appear to confer a sufficient degree of face validity (the phenomenological similarity between the phenotype of the experimental model and the human condition,^63^,^64^) to the experimental model presented herein^18^.

Pharmacological, neuroanatomical, and brain imaging studies indicate that neurological disturbances characterized by impaired motor function are associated with dysfunctions at the level of several brain structures and neurochemical pathways^65^. For example, TS generally involves abnormalities at the levels of the basal ganglia and several neurotransmitters^66^,^67^. While dopaminergic transmission has been associated with motor symptoms^23^, serotonin has been implicated both in motor inhibition and in obsessive-compulsive symptoms^25^,^65^. Based on this grounding, we predicted significant alterations in dopaminergic and serotonergic circuitry. The reduced serotonin concentrations measured in the prefrontal cortex of GAS treated mice support our predictions and may further extend the validity of the proposed experimental model. Thus, this structure has been shown to modulate perseverative responding both in clinical^68^ and preclinical^69^,^70^ investigations. Additionally, altered serotonin concentrations may partly account for the motor alterations observed in the open field and elevated 0-maze tests. The involvement of the serotonergic system in the control of repetitive behavior has been demonstrated through several experimental strategies. For example, several investigators demonstrated that the administration of selective 5-HT2 receptor agonists results in the exhibition of repetitive behaviors in mice^22^,^71^,^72^. Additionally, clinical data obtained through PET imaging indicate that TS patients exhibit elevated 5-HT2A receptor binding compared to healthy controls^73^. Furthermore, the observation that repeated GAS exposure altered brain neurochemistry in the cerebellum may provide additional support to the construct validity of the proposed model. Specifically, available literature suggests that the cerebellum is directly involved in spontaneous alternation^50^. For example, mutant mouse strains naturally exhibiting cerebellar atrophy have been shown to display deficits in spontaneous alternation^74^. Ultimately, experimental evidence indicates that serotonergic transmission may play a crucial role in modulating this domain in laboratory rodents^50^. Such evidence rests upon several studies conducted in rodents exposed to 5-HT receptor agonists^75^ or to a tryptophan deficient diet^76^. The role played by brain monoamines in modulating the behavioral phenotype is also partly substantiated by correlational evidence.

However, in contrast with our predictions and with previous literature^3^, we failed to observe significant alterations in dopaminergic transmission. With respect to rodent studies, we propose that differences in the species under analysis (rats in Refs. 3,21 and mice herein) and administration protocols may explain the discrepancy between our data and previous findings. Additionally, as cogently discussed by Buse and collaborators^23^ the relationship between dopamine and movement disorders is remarkably complex and, rather than being limited to differences in monoamine content, it may involve dopaminergic hyperinnervation, receptor sensitivity, and a dysfunction in the ratio between tonic and phasic activity^23^. Thus, future studies are needed to evaluate whether our experimental model presents analogous variations in the complex dopaminergic circuitry.

The autoimmune hypothesis behind our study poses that repeated peripheral exposure to GAS antigens would elicit an antibody-mediated response (see above), increase the permeability of the blood brain barrier^77^, facilitate the transition of peripheral antibodies in the central nervous system^78^, and ultimately result in autoimmune phenomena targeting brain areas involved in motor control and perseverative responding. These mechanisms were expected, in turn, to result in detectable inflammatory processes at the level of the cortico-striato-thalamo-cortical (CSTC) circuit^42^,^79^. To test this prediction, we adopted two complementary strategies. In a first batch of animals, we performed an *in vivo* magnetic resonance spectroscopy study in the striatum and prefrontal cortex of GAS treated and control mice. An independent set of mice were examined through immunohistochemistry to confirm that the behavioral, neurochemical, and metabolic abnormalities may be associated with antibody-mediated immune activation directed towards brain targets. In accordance with previous observations^19^,^20^, immunohistochemical assessment supported our original prediction by showing the presence of small inflammatory infiltrates and areas of diffuse microglia activation in all the examined GAS treated mice, in scattered areas of the rostral diencephalon controlling the behavioral domains addressed in the present study^50^. Additionally, in a subset of GAS treated mice, microglia nodules in normal appearing white matter, without evidence of inflammatory infiltrates, have been detected. This finding suggests that chronic inflammatory conditions induced by repeated GAS exposures and following brain inflammation, including microglia activation, may play a key role in the brain alteration observed in the current animal model probably by producing cytokines and neurotoxins, such as NO and/or glutamate, that may directly mediate neuroinflammation and tissue damage.

The elevated concentrations of glutamate and glutamine, detected in the striatum of GAS treated mice, constitute further indirect evidence that inflammatory processes may have occurred in response to repeated exposure. Based on the regulatory role exerted by glutamatergic projections on the basal ganglia^66^, elevated levels of glutamate have been suggested to play a role in several psychiatric disturbances^80^,^81^. Furthermore, inflammatory processes have been shown to increase glutamate concentrations in the basal ganglia in clinical studies conducted in a cohort of patients with hepatitis C^82^. The possibility that repeated GAS exposure resulted in inflammatory processes in TS-relevant brain areas is further substantiated by the elevated concentrations of lactic acid detected in the prefrontal cortex of GAS treated mice. Under physiological conditions, lactate constitutes the end product of anaerobic metabolism and, due to its physical properties, it is barely detectable in the brains of healthy experimental animals. Additionally, lactate concentrations vary depending on the specific brain area, and attain highest values in the striatum and smallest in cerebral cortex, with fluctuations due to nutritional state and anesthesia^83^,^84^. Due to its low level, lactate has been detected, in rodent brain cortex, only at magnetic fields higher than 9.4 T. Pathological conditions and inflammatory processes may affect the balance of anaerobic metabolism ultimately increasing lactate concentrations^85^. In accordance with these predictions, lactate concentrations were remarkably elevated in the prefrontal cortex of GAS treated mice and were not detected in control subjects. Since all subjects were submitted to the same dietary and anesthetic regimen, we suggest that the increase in lactate observed in the GAS group was due to the presence of inflammatory process. Although hypothetical, in accordance with Belanger and collaborators^86^, it may be tenable that the alterations observed in striatum and PFC reflect a functional interaction between these metabolites. Specifically, the authors proposed the existence of a mechanistic link (involving aerobic glycolysis) between glutamate release occurring in neurons, and extracellular lactate release dependent on astrocytes^86^. Finally, we failed to observe differences in lactate concentrations between GAS and control subjects in the striatum. Although hypothetical, we suggest that since lactate concentrations in the striatum are naturally higher compared to other brain structures^83^,^84^, potential ceiling effects may have masked underlying inflammatory processes.

In the present study, we strengthened the feasibility of an experimental model suited for the study of PANDAS. Additionally, the fact that the behavioral abnormalities are observed following repeated boosts, and not in response to the first immunization, indirectly supports the view that the exhibition of the pathological phenotype requires prolonged processes and high antibody levels to occur. This view is further supported by the observation that antibodies in serum were observed only in response to repeated boosts and not after the first immunization. This may also account for the fact that the development of the abnormal behavioral and neurological phenotype may entail gradual autoimmune responses directly targeting relevant neuroanatomical pathways. This hypothesis is in line with the immunohistochemical and MRS evaluations conducted in the brain areas putatively involved in the regulation of repetitive and perseverative behaviors. It is plausible that the GAS antigen/s responsible for the observed phenotype resides among the several proteins detected in treated animals. However, the identification of the precise antigen/s awaits future experiments wherein mice will be injected with single antigens in place of whole homogenates.

An additional limitation of the present study regards the timing of evaluation of the phenotype associated with streptococcus administration. As discussed in the introduction, the acronym PANDAS connotes a series of disturbances occurring in the pediatric population. Yet, both the present study and those conducted within the same theoretical framework^19^,^20^, have investigated young adult individuals. The principal reason for this limitation resides in technical constraints associated with the immunization protocol. To adequately mimic a disturbance associated with repeated exposure to streptococcus, the protocol necessitates several injections which, in turn, require some time to stimulate a complete immune response. Based on these constraints, and in accordance with previous literature, we deemed necessary to interspace the injections by three weeks. Thus, the effects of repeated exposures to streptococcus were assessed in young adult individuals. Nonetheless, the primary role played by an infantile immune system was addressed by beginning the injection protocol as early as PND 28 (i.e. before puberty). Future studies shall investigate whether the phenomena proposed herein can be observed in younger individuals. To address this possibility, it is tenable to propose a differential administration protocol entailing shorter between-boost intervals (e.g. two weeks) and an earlier timing of first injection (e.g. PND 21). Furthermore, to confirm that these processes are specifically dependent on exposures occurring during early maturational stages, future studies shall test the hypothesis that immunization procedures performed in adult individuals, are less efficient than identical procedures performed earlier in development.

Despite the aforementioned limitations, we believe that this study may inform future research from two complementary perspectives: (i) from a theoretical perspective, it gives support to the immune-mediated hypothesis of disorders like SC and TS, thereby allowing the analysis of novel prophylactic and therapeutic strategies aimed at preventing GAS colonization/infection in early infancy; (ii) from a preclinical perspective, the development of a mouse model possessing elevated construct and face validity may be leveraged to investigate the regulatory role exerted by the environment (e.g. psychosocial stress, difficult pregnancy, maternal smoking) in modulating the exhibition of symptoms. Moreover, a more detailed analysis of inflammatory events occurring in the brain of GAS mice may help clarifying the precise cell and molecular mechanisms involved in the neuropathology of the current experimental model.

## Additional Information

**How to cite this article**: Macrì, S. *et al*. Mice repeatedly exposed to Group-A *β*-Haemolytic Streptococcus show perseverative behaviors, impaired sensorimotor gating, and immune activation in rostral diencephalon. *Sci. Rep*. **5**, 13257; doi: 10.1038/srep13257 (2015).

## Supplementary Material

Supplementary Information
