# Publisher Correction: Mice repeatedly exposed to Group-A *β*-Haemolytic Streptococcus show perseverative behaviors, impaired sensorimotor gating, and immune activation in rostral diencephalon

**DOI:** 10.1038/s41598-020-69954-4

**Published:** 2020-08-03

**Authors:** Simone Macrì, Chiara Ceci, Martina Proietti Onori, Roberto William Invernizzi, Erika Bartolini, Luisa Altabella, Rossella Canese, Monica Imperi, Graziella Orefici, Roberta Creti, Immaculada Margarit, Roberta Magliozzi, Giovanni Laviola

**Affiliations:** 1 Sect. Behavioural Neuroscience, Dept. Cell Biology & Neuroscience, Istituto Superiore di Sanità, Viale Regina Elena, 299, I-00161 Roma, Italy; 2 IRCCS-Istituto di Ricerche Farmacologiche “Mario Negri”, Via G. La Masa 19, 20156 Milano, Italy; 3 Research Centre, Novartis Vaccines and Diagnostics, Via Fiorentina 1, 53100 Siena, Italy; 4 Sect. Molecular and Cellular Imaging, Dept. Cell Biology & Neuroscience, Istituto Superiore di Sanità, Viale Regina Elena, 299, I-00161 Roma, Italy; 5 Sect. Respiratory and Systemic Bacterial Diseases, Dept. of Infectious, Parasitic, and Immune-mediated Diseases, Istituto Superiore di Sanità, Viale Regina Elena, 299, I-00161 Roma, Italy; 6 Sect. Demyelinating and Inflammatory Diseases of the CNS, Dept. Cell Biology & Neuroscience, Istituto Superiore di Sanità, Viale Regina Elena, 299, I-00161 Roma, Italy

Correction to: *Scientific Reports* 10.1038/srep13257, published online 25 August 2015

In the original version of this Article, the author Martina Proietti Onori was incorrectly indexed. This error has now been corrected.

