# Supplementary material for: Mice repeatedly exposed to Group-A β-Haemolytic Streptococcus show perseverative behaviors, impaired sensorimotor gating, and immune activation in rostral diencephalon
Source: Sci Rep. 2015 Aug 25;5:13257. doi: 10.1038/srep13257 (PMC4548234; doi:10.1038/srep13257)
Supplement: Supplementary Information [file srep13257-s1.pdf]

Mice repeatedly exposed to Group-A β-Haemolytic Streptococcus show perseverative behaviors, impaired sensorimotor gating, and immune activation in rostral diencephalon

Simone Macrì, Chiara Ceci, Martina Proietti Onori, Roberto William Invernizzi, Erika Bartolini, Luisa Altabella, Rossella Canese, Monica Imperi, Graziella Orefici, Roberta Creti, Immaculada Margarit, Roberta Magliozzi, Giovanni Laviola

SUPPLEMENTARY INFORMATION

Magnetic resonance spectroscopy (MRS)

Animals were anaesthetised with isofluran 1.5–2.5% in O^2 1 l/min (Isoflo, Esteve Veterinaria, Milan, Italy) and maintained to the animal body temperature at 37.0 ± 0.1 °C by an integrated heating system. Gradient echo scout images were detected for accurate positioning of the head of the animal inside the magnet and spin echo sagittal anatomical images (TR/TE=3000/60 ms, 13 consecutive slices of 0.8 mm thickness, FOV=20 x 20 mm^2, matrix of 128 x 128, 2 averages) were acquired for positioning the voxel for the MRS study.

Localized shimming was performed up to water linewidths smaller or equal to < 12 Hz. Long TR and short TE parameters were selected in our pulse sequences, in order to be able to attribute any change in signal intensities to actual changes of metabolite levels. Nevertheless, T2 measurements were performed on water signal in order to identify any change due to treatment (a set of water localized spectra was acquired from the same voxel with the same parameters but TE ranging from 23 to 300 ms, 15 values). The water signal was suppressed by using the VAPOR pre-sequence composed by seven CHESS pulses with optimized flip angles and timing in order to have a reduced sensitivity to B1 variation and thus it is highly efficient also for surface coil; 256 averages were used to acquire metabolite spectra from PFC and STR. Unsuppressed water signal was acquired from the same voxel with the same parameters except for a reduced number of transients (4 instead of 256) and was used for metabolite quantification (assuming 79.9% for grey matter water content).

Spectra were analyzed using LCModel that calculates the best fit to the experimental spectrum as a linear combination of model spectra (spectra of metabolite solutions). Nine metabolites were included

in the basis set: creatine (Cr), glutamate (Glu), glutamine (Gln), myo-inositol (Ins), lactate (Lac), N-acetylaspartate (NAA), N-acetylaspartylglutamate (NAAG), phosphocreatine (PCr), and taurine (Tau). Spectra of lipids and macromolecules were also included in the basis set. Only those metabolites that were estimated to have Cramer-Rao lower bounds (CRLB) less than 20%, which corresponded to an estimated concentration error <0.2 μmol/g, were included into the quantitative analysis. In some cases, metabolites that have resonance overlapped or very close are also given as their sum. The signals due to inositol, glutamate and glutamine underwent J-coupling modulation with increasing TEs. However, the decreases in these signals due to J-coupling at TE=23 ms were automatically accounted for in the LCModel basis sets.

Table 1SM: Spatio-temporal and ethological parameters exhibited in the Elevated 0-Maze test after first injection (A) and second boost (B).

	PBS	GAS
**(A)**		
Number of entries in open arms	8.80 ± 1.76	8.60 ± 1.36
Time spent in open arms	44.77 ± 9.02	43.36 ± 8.32
Rearing frequency	17.30 ± 1.28	13.10 ± 0.98
Rearing duration (s)	31.43 ± 3.24	23.51 ± 2.65
Head dipping frequency	15.30 ± 1.69	14.00 ± 2.30
Head dipping duration (s)	39.48 ± 4.64	34.49 ± 7.15
Stretched attend posture frequency	14.80 ± 2.13	13.10 ± 1.48
Stretched attend posture duration (s)	40.19 ± 5.46	44.74 ± 5.54
Grooming frequency	1.60 ± 0.34	1.40 ± 0.27
Grooming duration (s)	3.44 ± 0.64	7.63 ± 2.31
**(B)**		
Number of entries in open arms	6.00 ± 1.15	6.00 ± 1.49
Time spent in open arms	35.54 ± 7.01	32.80 ± 6.36
Rearing frequency	14.50 ± 1.19	17.20 ± 2.08
Rearing duration (s)	22.39 ± 2.39	35.29 ± 5.56
Head dipping frequency	14.80 ± 2.13	12.40 ± 1.12
Head dipping duration (s)	30.71 ± 6.15	26.78 ± 2.47
Stretched attend posture frequency	23.00 ± 1.32	20.80 ± 1.18
Stretched attend posture duration (s)	68.52 ± 4.45	59.52 ± 3.99
Grooming frequency	1.80 ± 0.39	1.00 ± 0.26
Grooming duration (s)	10.50 ± 1.87	5.30 ± 1.35

Summary of data (mean ± SEM; N = 10, n = 20) observed in the Elevated 0-Maze following the first injection (A, week 5) and the second boost (B, week 11).

Table 2SM

Table 2SM: Concentrations of noradrenaline (NA), dopamine (DA) and its metabolites 3, 4-dihydroxyphenylacetic acid (DOPAC) and homovanillic acid (HVA) and its turnover (DOPAC/DA, HVA/DA) and of serotonin (5-HT), its metabolite 5-hydroxyindole acetic acid (5-HIAA) and its turnover (5-HIAA/5-HT).

	CTRL	GAS	*F* (df)	*p*
**(A)**				
NA	nd	nd	nd	nd
DA	3899.96 ± 350.81	3558.68 ± 197.92	0.72 (1,8)	0.42
DOPAC	1225.48 ± 154.63	1065.40 ± 71.10	0.88 (1,8)	0.38
HVA	440.15 ± 46.79	405.90 ± 22.45	0.43 (1,8)	0.53
DOPAC/DA	0.31 ± 0.02	0.30 ± 0.01	1.00 (1,8)	0.35
HVA/DA	0.11 ± 0.00	0.11 ± 0.00	nd	nd
5-HT	206.17 ± 17.02	210.39 ± 12.10	0.04 (1,8)	0.85
5-HIAA	248.94 ± 20.03	247.41 ± 10.76	0.01 (1,8)	0.95
5-HIAA/5-HT	1.21 ± 0.03	1.18 ± 0.06	0.10 (1,8)	0.76
**(B)**				
NA	nd	nd	nd	nd
DA	38.30 ± 5.35	36.15 ± 3.64	0.10 (1,8)	0.76
DOPAC	42.37 ± 3.62	45.74 ± 4.97	0.30 (1,8)	0.60
HVA	46.27 ± 2.97	41.17 ± 4.70	0.89 (1,8)	0.37
DOPAC/DA	1.15 ± 0.15	1.29 ± 0.15	0.40 (1,8)	0.55
HVA/DA	1.26 ± 0.15	1.14 ± 0.08	0.46 (1,8)	0.52
5-HT	825.37 ± 94.13	615.68 ± 45.96	4.01 (1,8)	0.04*
5-HIAA	504.77 ± 36.85	437.08 ± 25.27	2.31 (1,8)	0.17
5-HIAA/5-HT	0.67 ± 0.14	0.74 ± 0.10	0.06 (1,8)	0.82
**(C)**				
NA	nd	nd	nd	nd
DA	nd	nd	nd	nd
DOPAC	10.51 ± 0.65	11.77 ± 0.80	1.21 (1,8)	0.31
HVA	nd	nd	nd	nd
DOPAC/DA	nd	nd	nd	nd
HVA/DA	nd	nd	nd	nd
5-HT	85.64 ± 9.52	76.65 ± 3.63	0.73 (1,8)	0.42
5-HIAA	97.32 ± 8.65	102.87 ± 2.82	0.40 (1,8)	0.60
5-HIAA/5-HT	1.16 ± 0.09	1.35 ± 0.06	4.80 (1,8)	0.03*

(A) Levels of monoamine, their metabolites and their turnover (mean ± SEM; N = 10) in striatum of adult CTRL and GAS mice (ng/g tissue weight). * $p < 0.05$. nd = not detected.
(B) Levels of monoamine, their metabolites and their turnover (mean ± SEM; N = 10) in prefrontal cortex of adult CTRL and GAS mice (ng/g tissue weight). * $p < 0.05$. nd = not detected.
(C) Levels of monoamine, their metabolites and their turnover (mean ± SEM; N = 10) in cerebellum of adult CTRL and GAS mice (ng/g tissue weight). * $p < 0.05$. nd = not detected.

Table 3SM

Table 3SM: Correlations between monoamine concentrations in selected brain areas and behavior

	Correlation between 5-HT concentrations in hippocampus and behavior		Correlation between 5-HIAA/5-HT turnover in cerebellum and behavior	
	r^2 value	P value	r^2 value	P value
**Elevated 0-maze**				
Number of entries in open arms	0.014	0.74	0.002	0.89
Time spent in open arms (s)	0.023	0.67	0.049	0.53
Rearing frequency	0.073	0.45	0.2	0.19
Rearing duration (s)	0.001	0.94	0.52	0.018*
Head dipping frequency	0.1	0.35	0.21	0.17
Head dipping duration (s)	0.005	0.85	0.063	0.42
Stretched attend posture frequency	0.007	0.82	0.021	0.68
Stretched attend posture duration (s)	0.044	0.55	0.017	0.71
Grooming frequency	0.096	0.38	0.006	0.82
Grooming duration (s)	0.017	0.72	0.12	0.32
**Dowel test**				
Latency to fall	0.36	0.06	0.059	0.5
**Rotarod**				
Latency to fall	0.3	0.09	0.009	0.79
**Marble burying**				
N. of Marbles	0.045	0.55	0.001	0.92
**Open Field**				
Time spent in center	0.004	0.87	0.13	0.29
Time spent in periphery (s)	0.003	0.87	0.13	0.29
Frequency of crossings	0.5	0.021*	0.13	0.29
Rearing frequency	0.52	0.018*	0.095	0.38
Rearing duration (s)	0.6	0.008*	0.11	0.34
Grooming duration (s)	0.65	0.004*	0.09	0.4

Relationship (simple regression) between monoamines (5-HT concentration in hippocampus and 5-HIAA/5-HT turnover in cerebellum) and behavioral data observed in: Elevated 0-maze, Dowel test, Rotarod, Marble burying, and Open field. * $p < 0.05$.
